# Micronutrient loss in renal replacement therapy for acute kidney injury

**DOI:** 10.1186/cc14380

**Published:** 2015-03-16

**Authors:** W Oh, M Devonald, D Gardner, R Mahajan, D Harvey, A Sharman, B Mafrici, M Rigby, S Welham

**Affiliations:** 1 Nottingham University Hospitals NHS Trust, Nottingham, UK; 2 University of Nottingham, Sutton Bonington, UK; 3 University of Nottingham, UK

## Introduction

The prevalence of malnutrition in acute kidney injury (AKI) is high. Patients with AKI may require renal replacement therapy (RRT), which could result in loss of water-soluble micronutrients. Little is known about these losses in RRT and whether they differ between types of RRT. This study aims to quantify micronutrient losses during RRT in patients with AKI and to compare them in three different RRT modalities: continuous venovenous haemofiltration (CVVH), intermittent haemodialysis (IHD) and sustained low-efficiency diafiltration (SLEDf).

## Methods

A prospective observational study is being conducted at NUH. Thirty-three adult patients with AKI requiring RRT (13 IHD, 10 SLEDf, 10 CVVH) have been recruited. Samples of blood and RRT effluent were obtained at baseline, mid and end-session from each participant during their first RRT treatment. Samples were processed and stored at -80°C for subsequent analysis of amino acids by high-performance liquid chromatography and trace elements by inductively coupled mass spectrometry after derivatization from physiological fluids. Micronutrient losses were calculated by multiplying mass-corrected concentrations by total volume of RRT effluent, adjusted for baseline plasma concentrations and RRT dose. Data were analysed by restricted maximum likelihood estimating equations.

## Results

The total baseline plasma concentration of all standard amino acids was similar between IHD versus SLEDf groups (1,812 ± 517 vs. 2,675 ± 527 μmol/l, respectively) but were higher in the CVVH group (3,194 ± 564 μmol/l). RRT reduced the plasma concentration of amino acids in the SLEDf group (to 1,732 ± 529 μmol/l; *P *= 0.02), but had no effect in the IHD or CVVH groups (IHD; 1,853 ± 523, CVVH; 2,845 ± 512 μmol/l). The average, unadjusted loss of amino acids was significantly influenced by mode of RRT (IHD, 5.13 ± 3.1 vs. SLEDf, 8.21 ± 4.07 vs. CVVH, 18.69 ± 3.04 g; *P *< 0.01). The total baseline plasma concentration of trace elements was similar in the IHD, SLEDf and CVVH groups (3,797 ± 827, 3,667 ± 791, 3,642 ± 481 μg/l, respectively). By the end of the RRT session, the plasma concentration of trace elements had reduced (IHD, to 3,103 ± 827; SLEDf, to 2,805 ± 797; CVVH, to 3,433 ± 481 μg/l; *P *= 0.01). By the end of each RRT session, total losses of trace elements were estimated at IHD, 5,051 ± 2,312; SLEDf, 8,751 ± 2,421; CVVH, 11,258 ± 2,547 μg/l; *P *= 0.02 for treatment.

## Conclusion

Micronutrients are lost during RRT in AKI. The degree of micronutrient loss is influenced by the type of RRT used.

